# Frequency-Decoupled Dual-Stage Inverse Lithography Optimization via Hierarchical Sampling and Morphological Enhancement

**DOI:** 10.3390/mi16050515

**Published:** 2025-04-27

**Authors:** Jie Zhou, Qingyan Zhang, Haifeng Sun, Chuan Jin, Ji Zhou, Junbo Liu

**Affiliations:** 1 National Key Laboratory of Optical Field Manipulation Science and Technology, Chinese Academy of Sciences, Chengdu 610209, China; zhoujie23@mails.ucas.ac.cn (J.Z.); zhangqingyan22@mails.ucas.ac.cn (Q.Z.); hf_sun0804@163.com (H.S.); jinchuan17@mails.ucas.ac.cn (C.J.); zhouji@ioe.ac.cn (J.Z.); 2 State Key Lab of Optical Technologies on Nano-Fabrication and Micro-Engineering, Chinese Academy of Sciences, Chengdu 610209, China; 3 Institute of Optics and Electronics, Chinese Academy of Sciences, Chengdu 610209, China; 4 University of Chinese Academy of Sciences, Beijing 100049, China

**Keywords:** computational lithography, inverse lithography technology, dual-stage optimization, hierarchical sampling, morphological enhancement

## Abstract

Inverse lithography technology (ILT) plays a pivotal role in advanced semiconductor manufacturing because it enables pixel-level mask modifications, significantly enhances pattern fidelity, and expands process windows. However, traditional gradient-based ILT methods often struggle with the trade-off between imaging fidelity and mask manufacturability due to coupled optimization objectives. We propose a frequency-separated dual-stage optimization framework (FD-ILT) that strategically decouples these conflicting objectives by exploiting the inherent low-pass characteristics of lithographic systems. The first stage optimizes low-frequency (LF) components using hierarchical downsampling to generate a high-fidelity continuous transmission mask. This approach reduces computational complexity while refining resolution progressively. The second stage enforces manufacturability by exclusively adjusting high-frequency (HF) features through morphological regularization and progressive binarization penalties while maintaining the mask LF to preserve imaging accuracy. Our method achieves simultaneous control of both aspects by eliminating gradient conflicts between fidelity and manufacturing constraints. The simulation results demonstrate that FD-ILT achieves superior imaging quality and manufacturability compared to conventional gradient-based ILT methods, offering a scalable solution for advanced semiconductor nodes.

## 1. Introduction

As a pivotal process in integrated circuit (IC) fabrication, lithography transfers design patterns onto a photoresist-coated silicon wafer through precise optical exposure, forming intricate circuit structures via photochemical reactions [1]. The continued scaling driven by Moore’s law has resulted in increasingly pronounced diffraction effects during light–mask interactions in lithographic processes [2]. Higher-order diffraction components, which are essential for accurate pattern transfer, often exceed the collection capability of the projection lens due to their limited numerical aperture (NA). Consequently, these components fail to contribute to the imaging process, leading to significant discrepancies between the mask patterns projected onto the silicon wafer and the intended design pattern. This resolution-limiting phenomenon, the optical proximity effect (OPE), constitutes a fundamental limitation to pattern transfer fidelity and overall circuit performance, representing a critical manufacturing constraint in IC fabrication [3]. Optical proximity correction (OPC) is introduced as a pre-distortion compensation method during the mask design phase to mitigate the detrimental impact of OPE. OPC implements topology optimization to mask pattern edges through precisely calculated modifications, which are systematically generated through either empirical design rules or physics-based modeling [4].

While the conventional OPC demonstrates effectiveness in artifact mitigation, its localized correction schemes and Manhattan geometry correction constraints exhibit limited adaptability in dense layouts. These inherent constraints underscore the need for next-generation photomask optimization technology [5,6]. ILT provides more precise corrections through the mathematical reformulation of mask synthesis as an inverse optimization problem. Through pixel-level discretization and gradient-based optimization, ILT enables full-chip simultaneous optimization to attain superior pattern fidelity and process windows.

ILT algorithms are generally classified into two primary methodological approaches: gradient-based optimization and deep-learning-driven synthesis. The gradient-based approach formulates the mask design as a constrained optimization problem by minimizing the pixel-level discrepancy between the simulated resist profiles and target patterns via iterative gradient descent. This methodology systematically adjusts mask transmission values by computing derivatives of the cost function [7,8,9]. Conventional gradient-based ILT methods provide high design flexibility but have two limitations: they are geometrically complex and have high computational costs [10]. To address these limitations, researchers have proposed various optimization strategies and algorithms. In particular, the downsampling approach has proven effective in mask optimization by significantly reducing computational complexity [11,12,13,14]. For example, Lv et al. introduced a cascading multigrid algorithm for mask pattern optimization [11], and Sun et al. proposed a multilevel resolution method operating in the frequency domain [12]. Similarly, Chen et al. advanced the field using a bandwidth-constrained ILT method that explicitly incorporates the frequency-domain characteristics of lithographic imaging systems [13]. Additionally, momentum-based techniques and the utilization of graphics processing units (GPUs) have been proposed to further accelerate convergence [13,15], thereby enhancing optimization efficiency. To address issues regarding mask manufacturability, traditional gradient-based ILT methods often include various penalty terms within the objective function to enforce manufacturability constraints [16,17,18,19]. Furthermore, targeted optimization strategies have been developed to refine high-spatial-frequency mask components while preserving low-frequency features to improve mask manufacturability [20,21,22]. Although these methodologies have demonstrated significant advancements in mask optimization, they are still subject to a fundamental trade-off between imaging fidelity and manufacturability, which may lead to suboptimal solutions and local minima [18,19]. In contrast, deep-learning-based ILT methods employ neural networks to map target layouts to optimal mask patterns, thus facilitating rapid mask synthesis with reduced computational demands [23]. However, when trained on limited process window data, these approaches may yield locally optimal masks that do not satisfy physical constraints, limiting their applicability in production environments. Consequently, contemporary methodologies often utilize deep learning primarily for initialization in the subsequent gradient-based optimization steps to reduce the number of iterations required [24]. Despite these advancements, attaining an optimal balance among computational efficiency, imaging quality, and manufacturability remains a significant challenge.

To address the inherent challenges of achieving superior lithographic imaging quality and mask manufacturability in ILT, we present a physically motivated, dual-stage optimization framework that exploits the diffraction-limited properties of optical lithography systems. The core innovation lies in the frequency-domain decomposition of mask patterns, which permits independent optimization of low-frequency (LF) and high-frequency (HF) components in sequential stages. In the first stage, the optimization concentrates on extracting and refining the LF components that primarily govern imaging fidelity. Specifically, a hierarchical downsampling strategy combined with Gaussian filtering is employed to generate a high-fidelity continuous transmission mask (CTM) that preserves the essential features dictated by the cutoff frequency of the optical system. Furthermore, an adaptive gradient algorithm integrated with RMSProp is employed to accelerate the convergence process [25]. The subsequent stage refines the HF components by applying morphological operators and progressive binarization penalties to improve manufacturability while preserving the LF component. This decoupled strategy mitigates the inherent trade-offs encountered in conventional gradient-based ILT methods in which fidelity and manufacturability penalties are jointly embedded in a single cost function. The experimental results demonstrate that our frequency-decoupled dual-stage optimization framework achieves superior imaging quality and manufacturability compared to traditional ILT methods, offering a scalable solution for advanced semiconductor nodes.

## 2. Fundamentals of Gradient-Based ILT Optimization Framework

**Figure 1:**
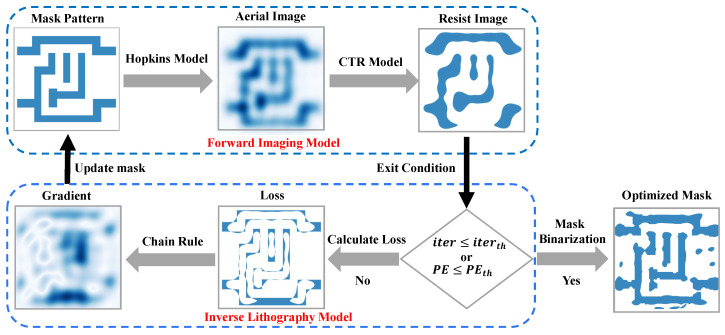
Conceptual illustration of traditional gradient-based ILT optimization framework.

Figure 1 illustrates the traditional gradient-based ILT workflow, comprising forward lithographic imaging modeling and inverse optimization. The forward model simulates resist image formation through optical and photochemical processes, while the inverse framework implements iterative mask refinement via gradient-guided updates. The optimization process terminates when the cost function convergence or reaches maximum iterations, followed by mask binarization.

### 2.1. Forward Lithography Imaging Model

The lithographic imaging procedure can be theoretically partitioned into two constituent models: an aerial image model and a resist model, as illustrated in Figure 1. The former model characterizes optical diffraction phenomena through the projection system, and the aerial image refers to the light intensity distribution on the photoresist surface, which is formed after the illumination passes through the mask and projection lens system. The imaging process is commonly described by the Hopkins equation [26]. To achieve more accurate simulations of the lithographic imaging process, vector effects must be considered for accurate simulation in high-NA systems [27]. The vector lithographic imaging process can be mathematically modeled as follows [28]:

(1) $$\begin{array}{cc} I(x,y)= & {\;\int \int \int \int \;}_{-\infty }^{\infty }TCC\left(f^{\prime },f^{\prime \prime };g^{\prime },g^{\prime \prime }\right)O\left(f^{\prime },g^{\prime }\right)O^{*}\left(f^{\prime \prime },g^{\prime \prime }\right)\\ \; & e^{-i2\pi [\left(f^{\prime }-f^{\prime \prime }\right)x+\left(g^{\prime }-g^{\prime \prime }\right)y]}\;df^{\prime }dg^{\prime }df^{\prime \prime }dg^{\prime \prime } \end{array}$$

In the above equation, I(x,y) denotes the intensity distribution across the wafer surface, O(f,g) represents the Fourier transform of the mask pattern, * denotes the complex conjunction operator, and $TCC(f^{\prime },f^{\prime \prime };g^{\prime },g^{\prime \prime })$ corresponds to the transfer cross coefficient (TCC). The TCC kernels encapsulate the optical characteristics of the lithographic system, including the illumination source and projection lens, and can be formulated as

(2) $$\begin{array}{cc} TCC_{ij}\left(f^{\prime },f^{\prime \prime };g^{\prime },g^{\prime \prime }\right)= & {\;\int \int \;}_{-\infty }^{+\infty }J\left(f^{\prime }+f_{c},g_{c}\right)H\left(f^{\prime }+f_{c},g^{\prime }+g_{c}\right)H^{*}\left(f^{\prime \prime }+f_{c},g^{\prime \prime }+g_{c}\right)\\ \; & M_{0i}\left(f^{\prime }+f_{c},g^{\prime }+g_{c}\right)M_{0j}^{*}\left(f^{\prime \prime }+f_{c},g^{\prime \prime }+g_{c}\right)E_{i}E_{j}^{*}df_{c}dg_{c} \end{array}$$

where $J(f^{\prime },g^{\prime })$ represents the intensity distribution of the partial coherent source, $H(f^{\prime },g^{\prime })$ denotes the pupil function of the projection lens, and $f_{c},g_{c}$ corresponds to the frequency offset. The term $M_{0}\left(f^{\prime },g^{\prime }\right)$ is the transfer matrix, while *E* indicates the electric field at the exit pupil. The TCC matrices, computed for different directions, can be summed to yield a unified TCC matrix.

(3) $$TCC=TCC_{xx}+TCC_{yx}+TCC_{zx}+TCC_{xy}+TCC_{yy}+TCC_{zy}$$

Subsequently, singular value decomposition (SVD) is employed to decompose the TCC matrix into a series of low-rank kernels, denoted by $\Phi_{k}$, each associated with an eigenvalue $\omega_{k}$. Only the first *K* terms are retained to enhance the computational efficiency of the lithography simulation. This approach, known as the Sum of Coherent Systems (SOCS) [29], can be formally expressed as

(4) $$TCC\left(f^{\prime },f^{\prime \prime };g^{\prime },g^{\prime \prime }\right)\approx \sum_{k=1}^{K}\omega_{k}\Phi_{k}\left(f^{\prime },g^{\prime }\right)\Phi_{k}^{*}\left(f^{\prime },g^{\prime }\right)$$

Drawing upon Equation (1), Equation (4), and convolution theorem [30], the aerial image intensity distribution on the wafer plane is calculated as follows:

(5) $$I\left(x,y\right)\approx \sum_{k=1}^{K}\omega_{k}{\left|\phi_{k}\left(x,y\right)⊗M\left(x,y\right)\right|}^{2}$$

where $\phi_{k}$ represents the inverse Fourier transform of $\Phi_{k}$, M(x,y) denotes the binary mask transmission function with values of 0 or 1, and ⊗ signifies the convolution operator.

The photoresist model defines patterned features transferred onto a silicon substrate [31]. For computational efficiency, we employ the constant threshold resist (CTR) model [7], which relies on a binary mechanism for pattern formation.

(6) $$R\left(x,y\right)=\left\{\begin{array}{cc} 1, & I\left(x,y\right)\geq I_{th}\\ 0, & \mathrm{otherwise} \end{array}\right.$$

where R(x,y)∈{0,1} represents the developed resist pattern and $I_{th}$ denotes the aerial image intensity threshold. For gradient-based optimization requiring differentiable models, the continuous resist response $R_{c}$ is approximated through a sigmoidal transfer function using the steepness factor $\theta_{r}$ as follows:

(7) $$R_{c}\left(x,y\right)=\frac{1}{1+\exp\left[-\theta_{r}\left(I\left(x,y\right)-I_{th}\right)\right]}$$

### 2.2. Inverse Lithography Optimization Framework

The Inverse Lithography Optimization (ILO) framework is devised to determine the optimal mask pattern, denoted as $M^{*}$, that minimizes the discrepancy between the target layout and the simulated resist image. This is achieved by establishing a cost function that quantifies the imaging performance, followed by the application of a gradient-based iterative refinement procedure to improve mask design, as illustrated in Figure 1. To ensure manufacturability, additional fabrication constraints are integrated into the cost function. Consequently, the ILO problem can be mathematically expressed as

(8) $$M^{*}=\arg\min_{M}\left[\lambda_{f}J_{f}\left(M\right)+\lambda_{r}J_{r}\left(M\right)\right]$$

where $J_{f}$ represents the fidelity metric and $J_{r}$ denotes the regularization penalty, each weighted by $\lambda_{f}$ and $\lambda_{r}$. The fidelity metric can be defined as the squared l2 norm of the discrepancy between the target pattern $R_{t}$ and resist pattern *R*:

(9) $$J_{f}\left(M\right)={∥R-R_{t}∥}_{2}^{2}$$

**Figure 2:**
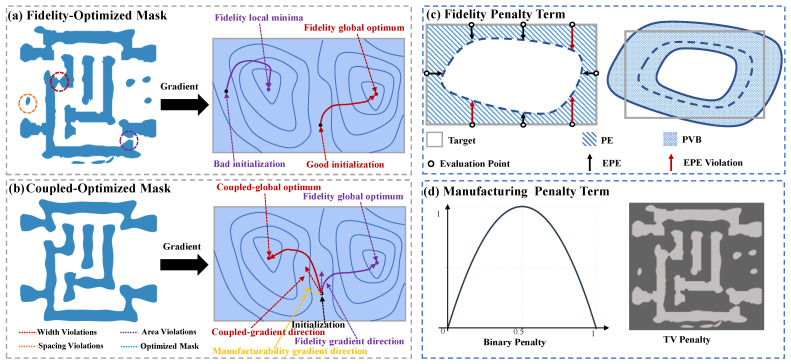
Traditional gradient-based ILT optimization trade-off between fidelity and manufacturability: (**a**) Fidelity-optimized mask and corresponding gradient, good fidelity but bad manufacturability. (**b**) Coupled-optimized mask and corresponding gradient, suboptimal fidelity and good manufacturability. (**c**) Fidelity penalty term: pattern error (PE), process variation band (PVB) and edge placement error (EPE). (**d**) Manufacturable penalty term: binary constraints and total variation (TV) constraints.

Meanwhile, the regularization term $J_{r}$ imposes constraints to ensure the feasibility of the mask production. These terms are integrated into the objective function to steer the optimization toward solutions that fulfill both design specifications and fabrication requirements. Typical regularization constraints encompass smoothness penalties and sparsity constraints [18,19,32,33], including total variation (TV) constraints and binary penalty terms, as shown in Figure 2.

The convex combination in Equation (8) inherently creates trade-offs between pattern fidelity and mask complexity, which may not always result in a globally optimal solution [18,19]. The optimization procedure progressively refines the mask pattern by minimizing the objective function using gradient-guided methods. However, the discrete binary constraints of mask patterning induce non-differentiable conditions, and mask transformation has been introduced to transform discrete parameters into a continuous domain [34]. Here, we adopt a sigmoidal transformation function:

(10) $$M\left(x,y\right)=\frac{1}{1+\exp\left[-\theta_{m}\left(M_{c}\left(x,y\right)-M_{th}\right)\right]}$$

where *M* denotes the binary mask, $M_{c}$ represents the CTM with values ranging continuously between 0 and 1, and $\theta_{m}$ dictates the gradient of the sigmoid function. Based on Equations (5),  (7),  (9), and  (10), the modified fidelity cost function is expressed as follows:

(11) $$J_{f}\left(M_{c}\right)=\sum_{x=1}^{N}\sum_{y=1}^{N}{\left\{\frac{1}{1+\exp\left[-\theta_{r}\left(\sum_{k=1}^{K}\omega_{k}{∥\phi_{k}⊗\frac{1}{1+\exp\left[-\theta_{m}\left(M_{c}\left(x,y\right)-M_{th}\right)\right]}∥}_{2}^{2}-I_{th}\right)\right]}-R_{t}\right\}}^{2}$$

The fidelity gradient can be derived as follows [35]:

(12) $$\begin{array}{cc} \frac{\partial J_{f}}{\partial M_{c}} & =2\theta_{r}\theta_{m}M_{c}⊙\left(1-M_{c}\right)⊙\\ \; & \;[\Phi ⊗\left(R_{c}⊙\left(1-R_{c}\right)⊙\left(R_{c}-R_{t}\right)⊙\left(M_{c}⊗\Phi^{*}\right)\right)\\ \; & \;+\Phi^{*}⊗\left(R_{c}⊙\left(1-R_{c}\right)⊙\left(R_{c}-R_{t}\right)⊙\left(M_{c}⊗\Phi \right)\right)] \end{array}$$

where $\Phi =\sum_{k=1}^{K}\omega_{k}\phi_{k}$, ⊙ denotes the Hadamard Product. Subsequently, the mask pattern $M_{c}^{k+1}$ is iteratively refined using gradient descent as follows:

(13) $$M_{c}^{k+1}=M_{c}^{k}-\eta \left(\lambda_{f}\frac{\partial J_{f}}{\partial M_{c}}+\lambda_{r}\frac{\partial J_{r}}{\partial M_{c}}\right)$$

where η represents the learning rate that controls the step size during each update, and the optimization procedure is halted when the cost function falls below a predefined threshold or when the maximum number of iterations is executed. Once convergence is achieved, the continuous mask undergoes a binarization process to generate the final binary mask pattern.

(14) $$M_{b}=\left\{\begin{array}{cc} 1, & M_{c}\geq M_{th}\\ 0, & M_{c}<M_{th} \end{array}\right.$$

$M_{th}$ represents the mask binarization threshold, which we set to 0.5 for the binary decision boundary during mask quantization. A fundamental challenge in gradient-based ILT optimization is the inherent trade-off between imaging fidelity and mask manufacturability, which arises from the coupled objectives within a single cost function, as illustrated in Figure 2. Figure 2a demonstrates a fidelity-optimized mask pattern that achieves superior imaging quality by exclusively minimizing the pattern error term; however, this approach yields structures with poor manufacturability characterized by fragmented features and complex geometries. In contrast, Figure 2b presents a jointly optimized mask that simultaneously addresses both fidelity and manufacturability constraints, resulting in enhanced fabrication feasibility but at the expense of degraded imaging performance. The specific optimization metrics employed in these approaches are depicted in Figure 2c, which illustrates the fidelity penalty terms, and Figure 2d, which shows the manufacturability constraints, consisting of binary and total variation regularization [7,18]. The binary penalty term enforces the mask to be binary, while the total variation penalty term enforces the mask to be smooth.

After optimization, mask performance evaluation requires quantitative metrics to assess both imaging fidelity and manufacturability. As illustrated in Figure 2c, fidelity assessment employs metrics including pattern error (PE), process variation band (PVB), and edge placement error (EPE). The PE metric quantifies imaging accuracy by computing the squared discrepancy between the target pattern $R_{t}$ and the simulated resist profile under nominal process conditions $R_{nom}$ as follows:

(15) $$\mathrm{PE}={∥R_{nom}-R_{t}∥}_{2}^{2}$$

PVB characterizes the manufacturing process robustness by computing the maximum contour separation area between the outermost contour $R_{out}$ and the innermost contour $R_{in}$ under different process conditions:

(16) $$\mathrm{PVB}={∥R_{out}-R_{in}∥}_{2}^{2}$$

The EPE quantifies lithographic fidelity by evaluating the critical dimension variations at predefined measurement points. Within a computational metrology framework, the algorithm examines the edge positions along the horizontal and vertical boundaries of the design and computes the Euclidean displacement D(x,y) between the printed pattern *R* and the target pattern $R_{t}$. A fidelity violation is recorded when the displacement exceeds the process tolerance threshold, $EPE_{th}$. Therefore, the violation indicator function performs binary classification as follows:

(17) $${\mathrm{EPE}}_{viol}\left(x,y\right)=\left\{\begin{array}{cc} 1, & D\left(x,y\right)\geq EPE_{th}\\ 0, & \mathrm{otherwise} \end{array}\right.$$

The composite EPE metric quantifies the cumulative spatial deviations observed across the measurement field:

(18) $$\mathrm{EPE}=\sum_{(x,y)\in \Omega }{\mathrm{EPE}}_{\mathrm{viol}}\left(x,y\right),\;\Omega =\Omega_{H}\cup \Omega_{V}$$

where $\Omega_{H}$ and $\Omega_{V}$ denote the horizontal and vertical edge evaluation points, respectively. The EPE metric is a comprehensive measure of the lithographic fidelity obtained by quantifying the deviations in edge positions across the target layout.

## 3. Frequency-Decoupled Dual-Stage ILT Optimization Algorithm

**Figure 3:**
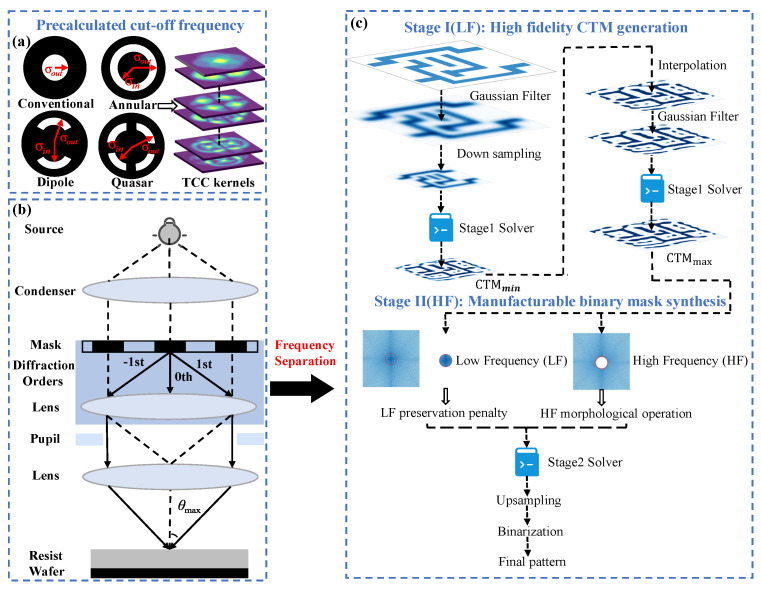
Frequency -separation dual-stage ILT optimization framework: (**a**) Illumination source and corresponding composite TCC kernel. (**b**) Lithography system’s intrinsic low-pass filtering properties. (**c**) Proposed frequency-decoupled dual-stage ILT workflow.

To address these challenges, we propose a dual-stage optimization framework based on frequency decomposition that leverages the inherent diffraction-limited characteristics of optical lithography systems, as illustrated in Figure 3. In this methodology, the mask pattern is partitioned into low-frequency (LF) and high-frequency (HF) components according to the cutoff frequency of the lithographic systems, with each element optimized sequentially. In the first stage, the LF component is refined to preserve optical fidelity, as detailed in Section 3.1. In the subsequent stage, manufacturability is enhanced by further refining the HF details of the CTM generated in the previous stage, as described in Section 3.2. This dual-stage strategy mitigates conflicting gradient effects, thereby ensuring both superior imaging quality and enhanced mask manufacturability.

### 3.1. High-Fidelity CTM Generation

The initial optimization stage focuses primarily on the LF components to guarantee high imaging fidelity. The corresponding pseudocode is shown in Algorithm 1. In this phase, a CTM is produced, enabling precise pattern transfer within the constraints imposed by the lithographic cutoff frequency. A hierarchical downsampling strategy, in conjunction with the sampling theorem, is employed to reduce computational complexity while preserving essential spatial-frequency information. This stage establishes a robust foundation for subsequent refinement through systematic dimensionality reduction by concentrating on LF elements that underpin the fundamental image formation process.

The proposed multiscale optimization framework systematically leverages the intrinsic low-pass characteristics of optical lithography systems, as shown in Figure 3. In particular, the optimization is limited to spatial frequencies below the diffraction-limited cutoff frequency, denoted by $f_{cut}$, as derived in [3].

(19) $$f_{cut}=\frac{\mathrm{NA}(1+\sigma_{\mathrm{out}})}{\lambda_{0}}$$

where NA denotes the numerical aperture, $\sigma_{\mathrm{out}}$ represents the maximum partial coherence factor as illustrated in Figure 3, and $\lambda_{0}$ corresponds to the illumination wavelength. To meet the Nyquist sampling criteria for the cutoff frequency of the system, the maximum allowable grid spacing $\Delta L_{max}$ is determined according to the following [13]:

(20) $$\Delta L_{max}\leq \frac{1}{2f_{cut}}$$

The initial grid spacing, $\Delta L_{0}$, is set to 1 nm. Subsequently, a cascadic sequence of grid spacings $\left\{\Delta L_{max},\ldots ,\Delta L_{1},\Delta L_{0}\right\}$ is established [11]. The downsampling strategy is initiated by applying a Gaussian filter, $H_{g}\left(u,v\right)$, to eliminate high-frequency components and mitigate spectral aliasing.

(21) $$H_{g}\left(u,v\right)=\exp\left(-\frac{u^{2}+v^{2}}{2\sigma^{2}}\right)$$

where σ denotes the standard deviation of the Gaussian kernel, which is set to $f_{cut}/2$ to guarantee effective low-pass filtering. The filtered mask pattern $M_{f}$ is subsequently obtained using

(22) $$M_{f}=F^{-1}\left(H_{g}\left(u,v\right)⊙F\left(M\right)\right)$$

where F and $F^{-1}$ represent the Fourier and inverse Fourier transforms, respectively. The use of Gaussian filtering enhances mask continuity and gradient uniformity relative to ideal low-pass filters, thereby yielding smoother optimization landscapes [36].
**Algorithm 1** High-fidelity CTM Generation**Require:** Target pattern $R_{t}$, optical parameters (NA, $\lambda_{0}$, $\sigma_{out}$), desired resolution n, maximum iterations $T_{\max}$ **Ensure:** Optimized CTM $M_{ctm}$
1:Initialize mask $M_{c}\leftarrow R_{t}$2:Compute base parameters:3:   $f_{cut}\leftarrow \frac{\mathrm{NA}(1+\sigma_{out})}{\lambda_{0}}$                                                                                                                                ▹ Cutoff frequency4:   $\Delta L_{max}\leftarrow 1/\left(2f_{cut}\right)$                                                                                                                                  ▹ Max grid spacing5:   $s_{0}\leftarrow \Delta L_{max}/\Delta L_{0}$                                                                                                                                              ▹ Scale factor6:Apply Gaussian filtering: $M_{f}\leftarrow F^{-1}\left(H_{g}⊙F\left(M_{c}\right)\right)$7:Downsample mask: $M_{f}^{i}\leftarrow \mathrm{downsample}\left(M_{f},s_{0}\right)$8:**for** i=0 **to** *n* **do**                                                                                                                               ▹ Multiscale processing9:    **while** $t<T_{\max}$ **do**                                                                                                                       ▹ Gradient optimization10:        Compute combined gradients: $\nabla J\leftarrow \lambda_{1}\nabla_{\mathrm{PE}}+\lambda_{2}\nabla_{\mathrm{PVB}}$11:        Low-pass filter gradient: $\nabla J_{\mathrm{LF}}\leftarrow H_{g}*\nabla J$12:        Update $M_{f}^{i}$ using RMSProp with $\nabla J_{\mathrm{LF}}$13:        t←t+1                                                                                                                             ▹ Update iteration counter14:    **end while**15:    **if** i<n **then**16:        Upsample: $M_{f}^{i+1}\leftarrow \mathrm{upsample}\left(M_{f}^{i},\times 2\right)$17:    **else**18:        $M_{ctm}\leftarrow M_{f}^{i}$                                                                                                                                                ▹ Final output19:    **end if**20:**end forreturn** $M_{ctm}$


The mask pattern is initially downsampled to the limiting resolution $\Delta L_{max}$ to facilitate efficient optimization, where the scale factor is defined as $s=\frac{\Delta L_{max}}{\Delta L_{0}}$. In contrast to conventional gradient-based ILT methods, we incorporate the PVB metric to ensure that the optimized mask exhibits robustness across diverse process conditions while omitting regularization terms. Consequently, the cost function is formulated as follows:

(23) $$M_{f}^{*}\left(sx,sy\right)=\arg\min_{M_{f}}\left[\lambda_{1}\mathrm{PE}\left(M_{f}\left(sx,sy\right)\right)+\lambda_{2}\mathrm{PVB}\left(M_{f}\left(sx,sy\right)\right)\right]$$

where $M_{f}\left(sx,sy\right)$ represents the low-pass CTM at scale *s*, and $\lambda_{1}$ and $\lambda_{2}$ denote the corresponding weighting factors. The gradient of PVB is computed in a manner analogous to Equation (12), with the distinction that the resist pattern is replaced by the outermost and innermost contours, $R_{out}$ and $R_{in}$, respectively.

(24) $$\frac{\partial \mathrm{PVB}}{\partial M_{f}}=2\left(R_{out}-R_{in}\right)⊙\left(\frac{\partial R_{out}}{\partial M_{f}}-\frac{\partial R_{in}}{\partial M_{f}}\right)$$

The derivative of $R_{\mathrm{out}}$ with respect to $M_{f}$ is derived as follows:

(25) $$\begin{array}{cc} \frac{\partial R_{out}}{\partial M_{f}}= & \theta_{r}\theta_{m}M_{f}⊙\left(1-M_{f}\right)⊙\\ \; & [\Phi_{out}⊗\left(R_{out}⊙\left(1-R_{out}\right)⊙\left(R_{out}-R_{t}\right)⊙\left(M_{f}⊗\Phi_{out}^{*}\right)\right)\\ \; & \;+\Phi_{out}^{*}⊗\left(R_{out}⊙\left(1-R_{out}\right)⊙\left(R_{out}-R_{t}\right)⊙\left(M_{f}⊗\Phi_{out}\right)\right)] \end{array}$$

$\frac{\partial R_{in}}{\partial M_{f}}$ can be analogously derived. Subsequently, the low-pass-filtered gradient is obtained by summing the PE and PVB gradients, each processed through a Gaussian filter:

(26) $$\frac{\partial J_{lf}}{\partial M_{f}}=F^{-1}\left(H_{g}\left(u,v\right)⊙F\left(\lambda_{1}\frac{\partial PE}{\partial M_{f}}+\lambda_{2}\frac{\partial PVB}{\partial M_{f}}\right)\right)$$

We then employed an adaptive gradient algorithm in conjunction with RMSProp to accelerate convergence. The adaptive gradient algorithm is a first-order optimization method that computes adaptive learning rates for individual parameters, thereby enabling accelerated convergence in complex optimization landscapes. At each iteration t, the algorithm updates the two-moment estimates, namely $m_{t}$ and $v_{t}$, based on the overall gradient $\frac{\partial J_{lf}}{\partial M_{f}}$. The first- and second-moment estimates are computed as follows:

(27) $$\left\{\begin{array}{c} m_{t}=\beta_{1}m_{t-1}+\left(1-\beta_{1}\right)\frac{\partial J_{lf}}{\partial M_{f}}\\ v_{t}=\beta_{2}v_{t-1}+\left(1-\beta_{2}\right){\frac{\partial J_{lf}}{\partial M_{f}}}^{2} \end{array}\right.$$

where $\beta_{1}$ and $\beta_{2}$ denote the decay rates, fixed at 0.9 and 0.999, respectively. To mitigate the bias inherent in the moment estimates, the bias-corrected estimates are computed as follows:

(28) $$\left\{\begin{array}{c} {\hat{m}}_{t}=\frac{m_{t}}{1-\beta_{1}^{t}}\\ {\hat{v}}_{t}=\frac{v_{t}}{1-\beta_{2}^{t}} \end{array}\right.$$

The parameter update equation is formally defined as follows:

(29) $$M_{c}^{t+1}=M_{c}^{t}-\eta \frac{{\hat{m}}_{t}}{\sqrt{{\hat{v}}_{t}}+\epsilon }$$

where η represents the learning rate and ϵ is a small constant, set to $10^{-8}$ to ensure numerical stability. This adaptive update mechanism dynamically modulates the learning rate for each parameter, thereby facilitating rapid convergence even in complex optimization landscapes.

Once the optimization process converges, the CTM corresponding to the maximum grid spacing $\Delta L_{max}$ is obtained. Subsequently, the optimized CTM is upscaled by a factor of two using bilinear interpolation to generate the mask pattern at the lower resolution. This process is iteratively repeated at each resolution until the desired resolution is achieved, with convergence at each stage accelerated by improved initial guesses. Ultimately, a CTM at the required resolution is produced, marking the conclusion of the first-stage optimization. This multiscale approach progressively refines the mask, yielding high-fidelity CTM with minimal computational overheads.

### 3.2. Manufacturable Binary Mask Synthesis

The subsequent optimization phase systematically refines the preliminary CTM through targeted manipulation of high-frequency (HF) components, thereby enhancing manufacturability while preserving critical imaging fidelity characteristics. Mask manufacturability compliance is rigorously quantified via mask rule check (MRC) criteria, which establish explicit constraints governing minimum feature spacing and area requirements within the mask pattern topology. This optimization procedure is comprehensively delineated in Algorithm 2. The methodology incorporates a low-frequency (LF) preservation regularization term to safeguard essential imaging attributes. Concurrently, morphological operations are systematically applied to ensure topological continuity, thus mitigating artifacts associated with discontinuities in mask geometry. Furthermore, a progressive binarization penalty function facilitates controlled intensity gradation, thereby enhancing convergence stability throughout the optimization process. This phase is characterized by exclusive focus on spectral mask manipulation through deliberate decoupling from the forward imaging model, yielding substantial improvements in computational efficiency. The resultant optimized patterns undergo threshold-based quantization, ultimately producing physically realizable binary photomask structures that satisfy stringent manufacturing requirements.
**Algorithm 2** Manufacturable Binary Mask Synthesis**Require:** Optimized CTM $M_{ctm}$, max iterations $T_{max}$ **Ensure:** Binary mask $M_{bin}$
1:Initialize $M\leftarrow M_{ctm}$ with combing kernel $\Phi =\sum_{k=1}^{K}\omega_{k}\phi_{k}$2:Set η←0.1, Δη←0.005, structural element *S*3:**for** t=1 **to** $T_{max}$ **do**4:    Compute gradients:5:       $\nabla J_{lf}\leftarrow \partial ∥F\left(\Phi \right)⊙\left(M-M_{ctm}\right){∥}^{2}/\partial M$                       ▹ Low-freq gradient6:       $\nabla J_{bin}\leftarrow 2M\left(1-M\right)\left(1-2M\right)$                                         ▹ Binary gradient7:    Update: $M\leftarrow M-\gamma \;\left[\left(1-\eta \right)\nabla J_{lf}+\eta \nabla J_{bin}\right]$8:    **if** tmod10=0 **then**9:        Apply morphological operations:10:           M←Opening(M,S)+Closing(M,S)−M11:        η←min(η+Δη,0.9)                                    ▹ Increase binary weight12:    **end if**13:**end for**14:Binarize: $M_{bin}\leftarrow I\left(M\geq 0.5\right)$ **return** $M_{bin}$


The CTM derived from the preceding stage is subjected to an HF refinement process to enhance manufacturability while preserving its critical LF components. Constrained optimization is formulated as follows:

(30) $$M_{\mathrm{bin}}^{*}=\arg\min_{M}\left[\left(1-\eta_{1}\right)J_{\mathrm{lf}}\left(M\right)+\eta_{1}J_{\mathrm{bin}}\left(M\right)\right]$$

Here, $J_{\mathrm{lf}}$ and $J_{\mathrm{bin}}$ represent the LF preservation penalty and the binary penalty terms of the mask, respectively. Parameter $\eta_{1}$ functions as a dynamic weighting factor that progressively decreases throughout the optimization. This gradual enforcement strategy mitigates abrupt transitions and prevents the algorithm from becoming trapped in local minima, thereby enhancing convergence stability.

The LF preservation penalty, which preserves the essential imaging characteristics owing to the low-pass nature of the lithography system, is defined as

(31) $$J_{\mathrm{lf}}\left(M\right)=∥F\left(\Phi \right)⊙[F\left(M\right)-F_{ctm}\left(M\right)]{∥}_{2}^{2}$$

where $F_{ctm}\left(M\right)$ denotes the Fourier transform of the optimized CTM from the previous stage and F(M) represents the Fourier transform of the mask in the current stage.

Incorporating a penalty term compels the mask values to converge toward the binary set {0,1} throughout the optimization process, thereby enhancing manufacturability.

(32) $$J_{\mathrm{bin}}\left(M\right)={∥M⊙\left(1-M\right)∥}_{2}^{2}$$

The progressive binarization strategy gradually increases the weight assigned to the binarization penalty term $\eta_{1}$ to 1 throughout the optimization process. This gradual weighting forces the grayscale values to converge incrementally toward the binary values, thereby avoiding an abrupt transition to a binary mask in the early stages. Consequently, this approach mitigates the tension between maintaining imaging fidelity and enforcing manufacturability constraints, ensuring a smooth transition that preserves critical LF features while progressively refining high-frequency details for optimal binarization.

We applied morphological operations to the mask [37], ensuring topological continuity and suppressing artifacts arising from fragmented mask patterns, as well as helping the optimization escape from the local optima. The erosion operation E(M,S) and dilation operation D(M,S) on mask *M* with respect to structural element *S* are formally defined as follows:

(33) $$\left\{\begin{array}{c} E\left(M,S\right)=\min_{(i,j)\in S}M\left(i,j\right)\\ D\left(M,S\right)=\max_{(i,j)\in S}M\left(i,j\right) \end{array}\right.$$

Then, the opening and closing operators can be derived as

(34) $$\left\{\begin{array}{c} O\left(M,S\right)=D\left(E(M,S),S\right)\\ C\left(M,S\right)=E\left(D(M,S),S\right) \end{array}\right.$$

Morphological operations are seamlessly incorporated into the mask synthesis process to enhance structural continuity and mitigate potential artifacts.

(35) $$M_{bin}=O\left(M_{bin},S\right)+C\left(M_{bin},S\right)-M_{bin}$$

The optimization procedure ends when either the cost function achieves a predefined threshold or a maximum number of iterations is reached. The final binary mask is subsequently derived by thresholding the optimized CTM, as specified in Equation (14).

## 4. Experiments

### 4.1. Experimental Setup

Our frequency-separation dual-stage ILT algorithm is implemented in Python 3.8 using the PyTorch 2.0 library. The experiments are conducted on a computer with a 3.8 GHz AMD Ryzen CPU and an NVIDIA GeForce RTX 4060 GPU. The proposed method is evaluated on simple and complex patterns using the ICCAD 2013 mask contest dataset [38]. The size of the mask is 2048 nm × 2048 nm, and the original resolution is 1 nm × 1 nm. An annular source with a wavelength of 193 nm is used. An exposure dose range of ±2% and a defocus range of ±25 nm are considered. The resist intensity threshold $I_{th}$ in Equation (6) is set to 0.225, the outermost contour is calculated under nominal focus and +2% nominal dose, and the innermost contour is estimated under a −25 nm defocus and −2% nominal dose. The EPE violation threshold, $EPE_{th}$, is set to 15 nm. The EPE is measured at the sample points on the horizontal and vertical edges of the target pattern every 40 nm. The resist sensitivity, $\theta_{r}$, and mask sensitivity, $\theta_{m}$, are set to 4. The standard deviation σ is set to 3. The weights of Equation (23) are set to 1. The weights of Equation (30) are set to $\eta_{1}=0.9$. The maximum number of scales is set to 8. The maximum number of iterations is set to 100.

### 4.2. Different Cost Function Simulation Result Analysis

**Figure 4:**
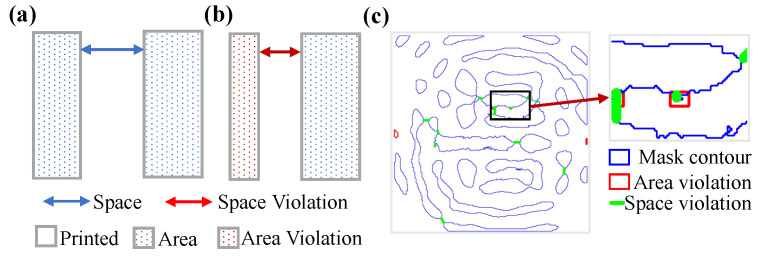
Mask rule check (MRC) definitions: (**a**) MRC satisfied; (**b**) MRC violations; (**c**) fidelity-only cost function-optimized mask and corresponding MRC violations.

To evaluate the manufacturability of the final binary mask, we performed mask rule check (MRC) to quantitatively assess area and spacing violations [39]. These evaluations enforce a set of geometric constraints that ensure the mask complies with established manufacturability standards, as depicted in Figure 4. In our experiments, the minimum spacing requirement is defined as 40 nm and the minimum area is specified as 1600 nm^2^.

**Figure 5:**
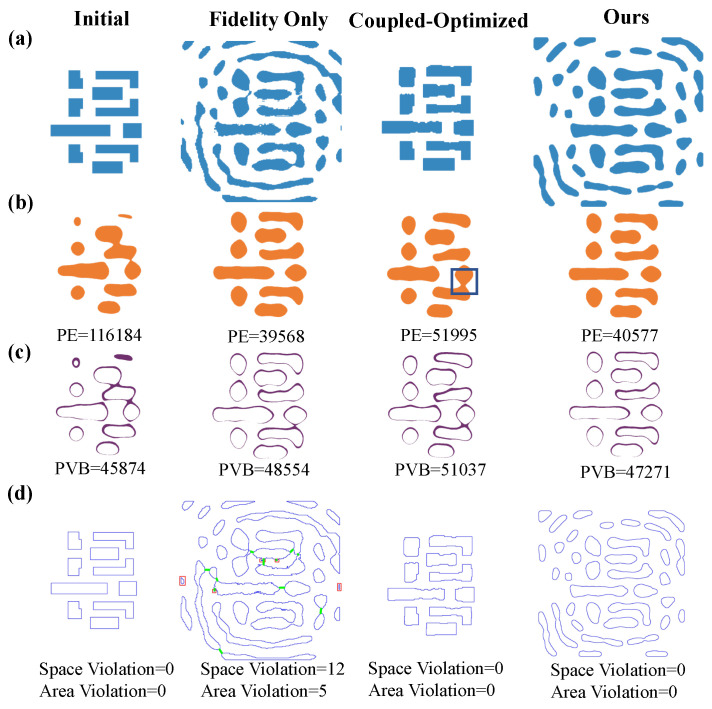
Simulation results for different cost functions: (**a**) different mask synthesis methods; (**b**) PE metrics; (**c**) PVB metrics; (**d**) MRC violations. The blue box in (**b**) indicates the bridge region.

As shown in Figure 5, the influence of different cost function formulations is clearly manifested across a range of optimization scenarios. The fidelity-only approach, which exclusively prioritizes imaging quality, effectively reduces the PE metric from 116,184 to 39,568. However, this improvement comes at the cost of significant MRC violations, as evidenced in Figure 4. In contrast, the integration of manufacturing penalties into the cost function successfully eliminates MRC violations, albeit with a trade-off that results in an elevated PE. This underscores an intrinsic tension between imaging fidelity and manufacturability. The proposed FD-ILT framework addresses this challenge through a dual-stage optimization strategy, achieving a PE of 40,577 while ensuring full MRC compliance. These results substantiate the efficacy of the FD-ILT framework in simultaneously enhancing imaging fidelity and adhering to manufacturability requirements.

### 4.3. Simulation Results for Mask Fidelity Analysis

**Figure 6:**
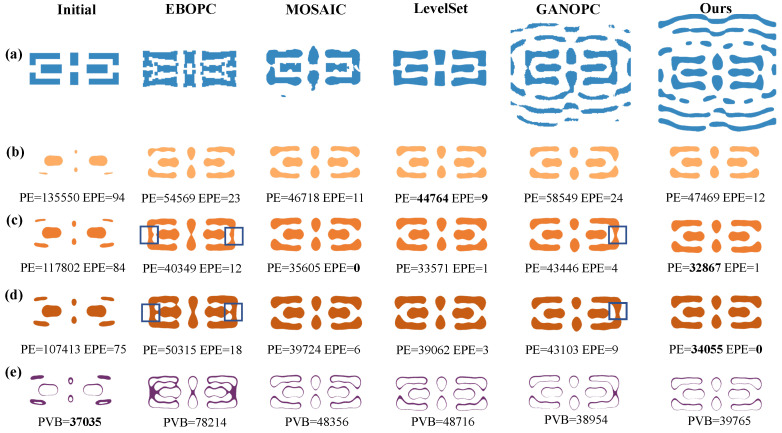
Simulation results for simple patterns: (**a**) Optimized mask for different methods. (**b**) Resist image under minimal conditions. (**c**) Resist image under nominal conditions. (**d**) Resist image under maximum conditions. (**e**) PVB metrics. The blue box in (**c**,**d**) indicates the bridge region.

Figure 6 presents a comparative analysis of simulation results for simple patterns evaluated under varying process conditions. The top row displays optimized masks produced by different algorithms, while the subsequent rows illustrate the corresponding resist images under minimum, nominal, and maximum process conditions, with the final row depicting the PVB. The initial target pattern, shown in the first column, exhibits pronounced distortion under nominal conditions, as quantified by a PE of 116,184 nm^2^ and an EPE of 84. The edge-based optical proximity correction (EBOPC) method [40], depicted in the second column, reduces the PE to 40,349 nm^2^ and the EPE to 12 relative to the initial mask. This improvement enhances imaging quality; however, persistent bridge defects between pattern edges remain unresolved. In the third column, the MOSAIC method [35], which emphasizes fidelity optimization, outperforms conventional OPC techniques by achieving a PE of 35,605 nm^2^ and an EPE of 0. Despite this superior imaging fidelity, the optimized mask pattern compromises manufacturability, limiting its practical applicability. The level-set approach [15], presented in the fourth column, further improves imaging quality, yielding a PE of 38,927 nm^2^ and an EPE of 1, reflecting effective pattern fidelity under nominal conditions. Similarly, the generative adversarial network-based GANOPC method [41], shown in the fifth column, reduces the PE to 43,446 nm^2^ and the EPE to 4 compared to the initial pattern. However, its performance is undermined by process instability, as evidenced by bridge defects under nominal and maximum conditions, attributable to inadequate incorporation of physical constraints. The proposed FD-ILT methodology in the final column achieves optimal performance with a PE of 32,867 nm^2^ and an EPE of 1 under nominal conditions, demonstrating superior imaging quality characterized by enhanced contour smoothness and minimized pattern distortion compared with other methods. The PVB metric of our method is 39,765 nm^2^, which indicates the robustness of the mask pattern under process variations.

**Figure 7:**
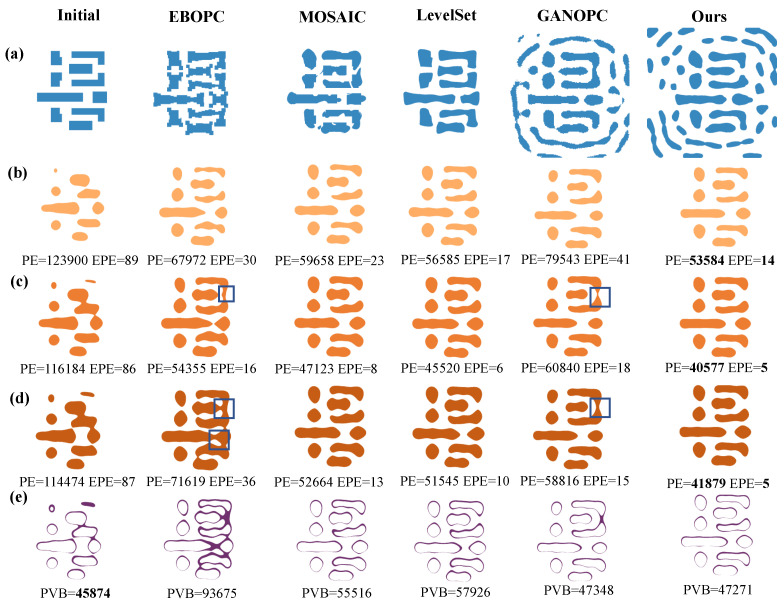
Simulation results for complex patterns: (**a**) Optimized mask for different methods. (**b**) Resist image under minimal conditions. (**c**) Resist image under nominal conditions. (**d**) Resist image under maximum conditions. (**e**) PVB metrics. The blue box in (**c**,**d**) indicates the bridge region.

To further evaluate our FD-ILT framework, we conducted experiments on a complex mask pattern, as shown in Figure 7. The first row denotes various optimized masks generated by different algorithms, and the subsequent three rows illustrate the resist images under different process conditions. The baseline initial pattern demonstrates substantial lithographic instability, as evidenced by the excessive PE values of 123,900 nm^2^, 116,184 nm^2^, and 114,474 nm^2^. The EBOPC method exhibits inherent limitations in both image quality and process variation tolerance, primarily because of its heuristic edge fragmentation approach. The PE metrics for different conditions are 67,972 nm^2^, 54,355 nm^2^, and 71,619 nm^2^, while the EPE metrics are 30, 16, and 36, respectively, and the PVB metric is 93,675 nm^2^, indicating limited process variation tolerance. Although the MOSAIC method achieved enhanced pattern fidelity through rigorous pixel-level optimization, the PE metrics are reduced by 12.2%, 24.3%, and 26.4%, respectively, whereas the EPE metrics are significantly reduced by 23.3%, 50%, and 63.8%, respectively, and the PVB metric is reduced by 40.7%. The level-set method implements an implicit boundary representation via higher-dimensional function evolution, thereby achieving superior mask regularity through intrinsic smoothness constraints. The average PE and EPE metrics are reduced by 3.6% and 25%, respectively, compared to the MOSAIC method, with the PVB slightly increasing from 55,516 nm^2^ to 57,926 nm^2^. The GANOPC exhibits suboptimal performance compared to other methods, with the average PE and EPE metrics increasing by 38.7% and 124.2% compared to the level-set method, while the PVB metrics are relatively low (47,248 nm^2^) compared to other methods. The proposed FD-ILT method achieves the best fidelity compared to other methods, with the smallest PE and EPE metrics under different process conditions, reducing the average PE and the average EPE by 61.6% and 90.9%, respectively, compared to the target pattern, while the PVB metrics are slightly increased (3%) compared to the original pattern. The EBOPC and GANOPC methods suffer from resist bridge defeats, similar to the simple pattern.

### 4.4. Simulation Results for Mask Manufacturability Analysis

**Figure 8:**
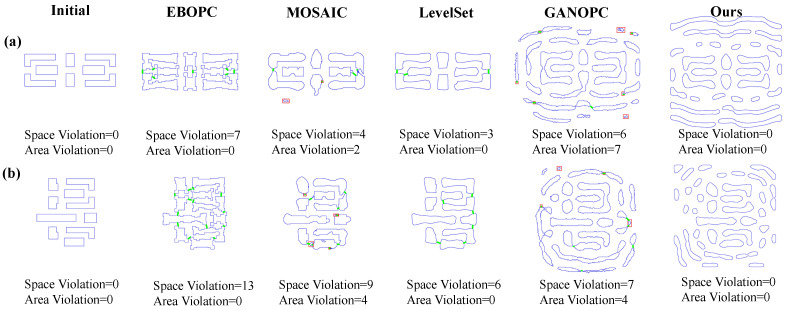
Simulation results for manufacturability: (**a**) MRC violations for simple patterns; (**b**) MRC violations for complex patterns. The blue contour is the mask pattern, the red box denote the area violation, the green line denotes the spacing violation.

Figure 8 illustrates the manufacturability analysis of the optimized mask patterns for both simple and complex patterns. The top row presents the optimized mask contours and MRC violations for the simple pattern, whereas the subsequent rows depict those for the complex pattern. Each column presents the target pattern, optimized mask patterns generated by EBOPC, MOSAIC, LevelSet, GANOPC, and the proposed FD-ILT method. The original pattern is not violated. The EBOPC method adjusts the pattern edges and violates the spacing constraints without area constraints, resulting in space violations of 7 and 13 for simple and complex patterns, respectively. The MOSAIC method exhibits poor manufacturability owing to the lack of manufacturability constraints, with a simple pattern having four minimal space violations and two minimal area violations, while the complex pattern has nine minimal space violations and four minimal area violations. The level-set method has better manufacturability by optimizing the mask boundaries, with three and six minimal space violations for the simple and complex patterns, respectively. The GANOPC method exhibits the poorest manufacturability, with six minimal space violations and seven minimal area violations for the simple pattern, and seven minimal space violations and four minimal area violations for the complex pattern, owing to the generation of masks without physical constraints. The proposed FD-ILT method achieves superior manufacturability by optimizing the mask pattern manufacturability separately from fidelity. The simple pattern and complex pattern have 0 minimal space violations and 0 minimal area violations. The proposed method utilizes morphological operations to ensure topological continuity while avoiding space and area violations.

## 5. Conclusions

This paper proposes a dual-stage Inverse Lithography Optimization framework that decouples imaging quality and manufacturability by leveraging the inherent low-pass characteristics of lithographic systems. In the first stage, hierarchical sampling reduces the computational complexity by adaptively downsampling mask gradients, which enables the efficient generation of high-fidelity CTM accelerated by RMSProp with momentum. In the subsequent stage, high-frequency details are refined via gradient-guided optimization complemented by morphological operations and a progressive binarization penalty. This dual-stage approach yields manufacturable binary masks that preserve critical imaging features while satisfying fabrication constraints. The experimental results demonstrate the framework’s superior performance in imaging fidelity, manufacturability, and computational efficiency, highlighting its potential for next-generation lithography applications.

## Data Availability

All data are included in the study.
